# Nintedanib Regulates GRK2 and CXCR2 to Reduce Neutrophil Recruitment in Endotoxin-Induced Lung Injury

**DOI:** 10.3390/ijms22189898

**Published:** 2021-09-13

**Authors:** Vincent Yi-Fong Su, Wei-Chih Chen, Wen-Kuang Yu, Huai-Hsuan Wu, Hao Chen, Kuang-Yao Yang

**Affiliations:** 1 School of Medicine, College of Medicine, National Yang Ming Chiao Tung University, No. 155, Sec. 2, Linong St, Taipei 11221, Taiwan; bsbipoke@hotmail.com (V.Y.-F.S.); wcchen2@vghtpe.gov.tw (W.-C.C.); wkyu2@vghtpe.gov.tw (W.-K.Y.); 2 Department of Internal Medicine, Taipei City Hospital, Taipei City Government, Taipei 10364, Taiwan; 3 Department of Chest Medicine, Taipei Veterans General Hospital, No. 201, Sec. 2, Shi-Pai Road, Taipei 11217, Taiwan; purplewings0401@gmail.com (H.-H.W.); asura811218@gmail.com (H.C.); 4 Institute of Emergency and Critical Care Medicine, School of Medicine, National Yang Ming Chiao Tung University, Taipei 11221, Taiwan; 5 Cancer Progression Research Center, National Yang Ming Chiao Tung University, Taipei 11221, Taiwan

**Keywords:** nintedanib, chemotaxis, GRK2, p38 MAPK, CXCR2, acute lung injury, neutrophil chemotaxis

## Abstract

The role of nintedanib, a multiple tyrosine kinase inhibitor, in the treatment of sepsis-induced acute lung injury (ALI) remains unclear. Lipopolysaccharide (LPS), also known as endotoxin, has been used to induce ALI. The goal of this study was to assess the effect of nintedanib in attenuating the histopathological changes of LPS-induced ALI. Nintedanib was administered via oral gavage to male C57BL/6 mice 24 h and 10 min before intratracheal endotoxin instillation. Lung histopathological characteristics, adhesion molecule expression, and the regulatory signaling pathways of neutrophil chemotaxis were analyzed after 24 h. We found that nintedanib significantly reduced histopathological changes and neutrophil recruitment in LPS-induced ALI. The number of neutrophils in bronchoalveolar lavage fluid (BALF) was reduced in nintedanib-treated relative to untreated mice with ALI. Nintedanib mediated the downregulation of the chemotactic response to LPS by reducing the expression of adhesion molecules and the phosphorylated p38:total p38 mitogen-activated protein kinase (MAPK) ratio in the lungs of mice with ALI. Nintedanib also reduced the expression of lymphocyte antigen 6 complex locus G6D (Ly6G) and very late antigen 4 (VLA-4) in BALF neutrophils and mediated the downregulation of chemokine (C-X-C motif) receptor 2 (CXCR2) and upregulation of G protein-coupled receptor kinase 2 (GRK2) activity in peripheral blood neutrophils in mice with LPS-induced ALI. Nintedanib improved the histopathological changes of LPS-induced ALI by reducing neutrophil chemotaxis. These effects were mediated by the inhibition of adhesion molecules via the activation of GRK2 and the inhibition of p38 MAPK and CXCR2.

## 1. Introduction

Acute lung injury (ALI), and its most severe form, acute respiratory distress syndrome (ARDS), are life-threatening diseases in critically ill patients. The pathological changes caused by ALI/ARDS result in severe hypoxemic respiratory failure and mortality. ALI is characterized by the recruitment of neutrophils into the alveolar space, interstitial edema, and endothelial and epithelial injury [1]. Neutrophils, the inflammatory cells that respond earliest to sepsis, are recruited following an inflammatory stimulus in sepsis-induced ALI.

Chemokine (C-X-C motif) receptor 2 (CXCR2), a seven-transmembrane G protein-coupled receptor of human CXC chemokines, is expressed in human polymorphonuclear leukocytes (PMNs). CXCR2 belongs to the CXCR family and is the major receptor of chemotactic factors that mediate migration [2]. In models of sepsis-induced ALI, CXCR2 mediates the migration of neutrophils into the lung. Macrophage-inflammatory protein 2 (MIP-2), a cytokine belonging to the CXC chemokine family and among the most common chemotactic factors, responds to lipopolysaccharides (LPSs) by activating neutrophil migration to sites of inflammation or infection, including the lung in patients with sepsis-induced ALI [3]. Monocytes, macrophages, and neutrophils secrete MIP-2, which modulates neutrophil chemotaxis by activating CXCR2 [4]. G protein-coupled receptor kinases (GRKs) were originally identified as key regulators of G protein-coupled receptor function. GRK2 is highly expressed on neutrophils and appears to be an important regulator of the migratory response during inflammation [5,6]. Recent data indicate that the inhibition of GRK2 can increase CXCR2 activity and decrease CXCR2 resistance to phosphorylation, desensitization, and internalization [7].

Nintedanib is a small-molecule tyrosine kinase inhibitor that blocks the action of platelet-derived growth factor receptor, the vascular endothelial growth factor receptor and the fibroblast growth factor receptor. Its use as an antifibrotic drug has been investigated, and it has been approved for the treatment of idiopathic pulmonary fibrosis. Our previous work indicated that nintedanib attenuates bleomycin-induced pulmonary fibrosis in mice via the activation of GRK2 expression [8]. However, the role of nintedanib in the treatment of sepsis-induced ALI is not fully understood. Since neutrophil chemotaxis has been implicated in ALI induced by LPS, we examined the protective effect of nintedanib on ALI induced by LPS. In the present report, we describe our investigation of the effects of nintedanib via the moderation of neutrophil chemotaxis in a mouse model of LPS-induced ALI.

## 2. Results

### 2.1. Effects of Nintedanib on the Histopathological Features and Fibrosis of LPS-Induced ALI

**Figure 1:**
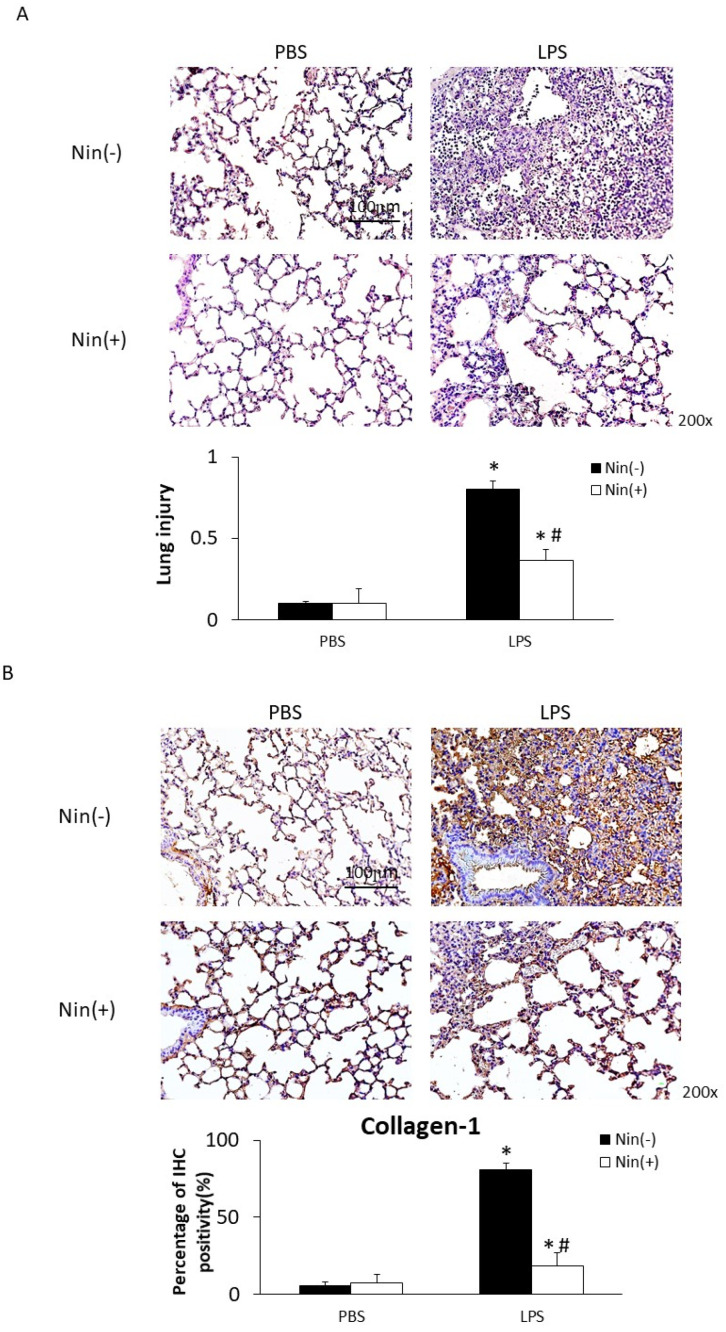
Oral nintedanib (Nin) administration improved histological features and fibrosis in mice with lipopolysaccharide (LPS)-induced acute lung injury (ALI). (**A**) Hematoxylin and eosin staining revealed minimal histopathological abnormalities in control mice injected intratracheally with phosphate-buffered saline (PBS). Oral nintedanib administration attenuated pathological changes in lung injury in mice with LPS-induced ALI. Mice with ALI treated with nintedanib had significantly lower lung injury scores than did mice with untreated ALI. (**B**) Immunohistochemical staining showed that intratracheal injection of LPS significantly increased the expression of collagen-1. The oral administration of nintedanib inhibited the fibrotic changes in mice with LPS-induced ALI. Data are means ± standard deviations. * *p* < 0.05 vs. control, # *p* < 0.05 vs. Nin (−); *n* = 6 per group.

Intratracheal injection of LPS resulted in ALI, characterized by interstitial and alveolar edema with the accumulation of neutrophils, macrophages, and red blood cells in the alveolar spaces, and interstitial collagen deposition. Histological evaluation of lung sections showed that nintedanib treatment significantly reduced the severity of lung injury; accordingly, it also significantly reduced lung injury scores (0.37 vs. 0.80, *p* < 0.05, Figure 1A). Nintedanib significantly reduced collagen deposition (collagen-1 staining: 18.2% vs. 80.6%, *p* < 0.05, Figure 1B).

### 2.2. Effects of Nintedanib on Neutrophil Migration in the Lung

**Figure 2:**
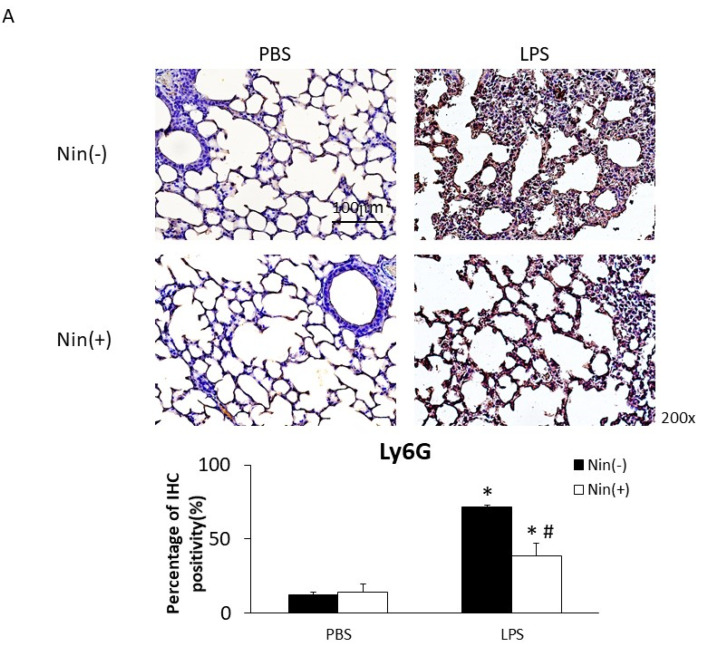

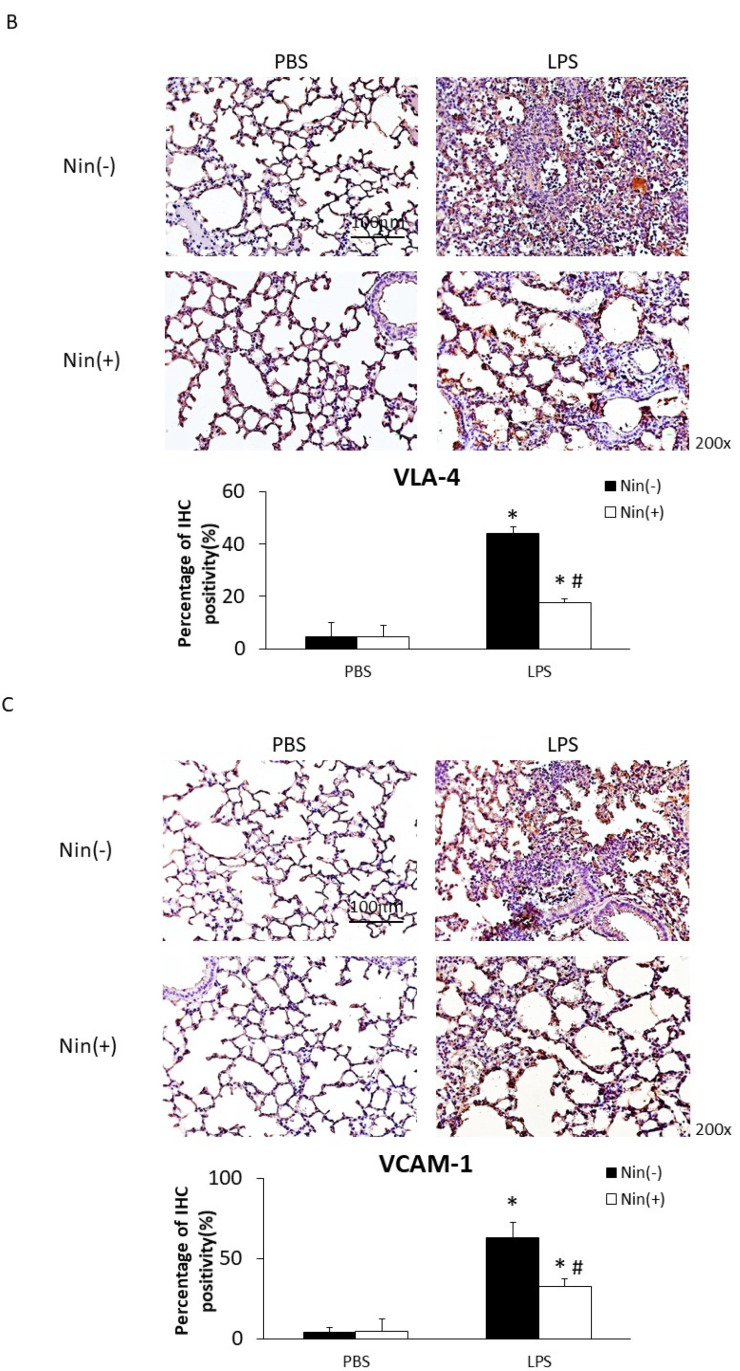
Nintedanib (Nin) administration restored the expression of the neutrophil marker Ly6G, VLA-4 and VCAM-1 in the lungs of mice with LPS-induced ALI. (**A**–**C**) Intratracheal injection of LPS significantly increased the expression of Ly6G, VLA-4, and VCAM-1 in the lungs of mice with ALI compared with control mice. Nintedanib administration reversed these changes. Data are means ± standard deviations. * *p* < 0.05 vs. control, # *p* < 0.05 vs. LPS; *n* = 6 per group. PBS, phosphate-buffered saline; IHC, immunohistochemical stains.

The intratracheal instillation of LPS resulted in a significant increase in PMN accumulation in the lungs relative to the control, as demonstrated visually by Ly6G staining (reflecting positivity for neutrophils; 71.6% vs. 12.3%, *p* < 0.05). Nintedanib significantly reduced neutrophil accumulation (Ly6G staining: 38.4% vs. 71.6%, *p* < 0.05) after LPS injection (Figure 2A).

Immunohistochemical (IHC) staining of lung tissue showed that the expression levels of VLA-4 and VCAM-1 (the counter-receptor of VLA-4) had increased significantly 24 h after LPS-induced ALI compared with the control (VLA-4, 4.7% vs. 44.1%; VCAM-1, 4.0% vs. 62.9%; both *p* < 0.05). Nintedanib significantly reduced this expression (VLA-4, 17.7% vs. 44.1%; VCAM-1, 32.7% vs. 62.9%; both *p* < 0.05; Figure 2B).

**Figure 3:**
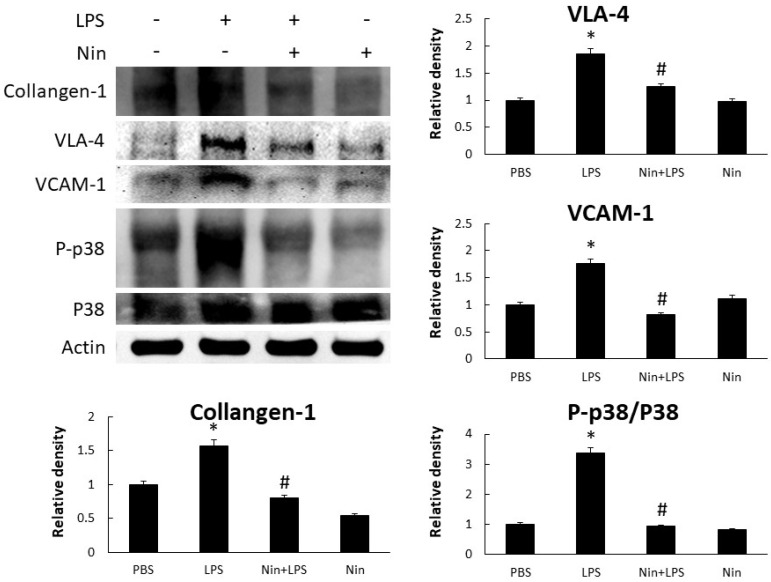
Nintedanib (Nin) administration restored the phosphorylated-p38:p38 ratio (P-p38/p38) to inhibit neutrophil migration and fibrotic changes in mice with LPS-induced ALI. Western blots confirmed that the intratracheal injection of LPS significantly increased the expression of VLA-4 and VCAM-1 and collagen-1, and P-p38/p38, in the lungs of these mice. Oral nintedanib administration reversed these changes. Data are means ± standard deviations. * *p* < 0.05 vs. control, # *p* < 0.05 vs. LPS; *n* = 6 per group. PBS, phosphate-buffered saline.

Western blot analysis confirmed that LPS induced the expression of collagen-1, VLA-4, VCAM-1, and phosphorylated-p38:p38 ratio (P-p38/p38), and that nintedanib restored these changes, in the lung tissue of mice with ALI (Figure 3).

### 2.3. Effects of Nintedanib on Neutrophil Accumulation in the Lung

**Figure 4:**
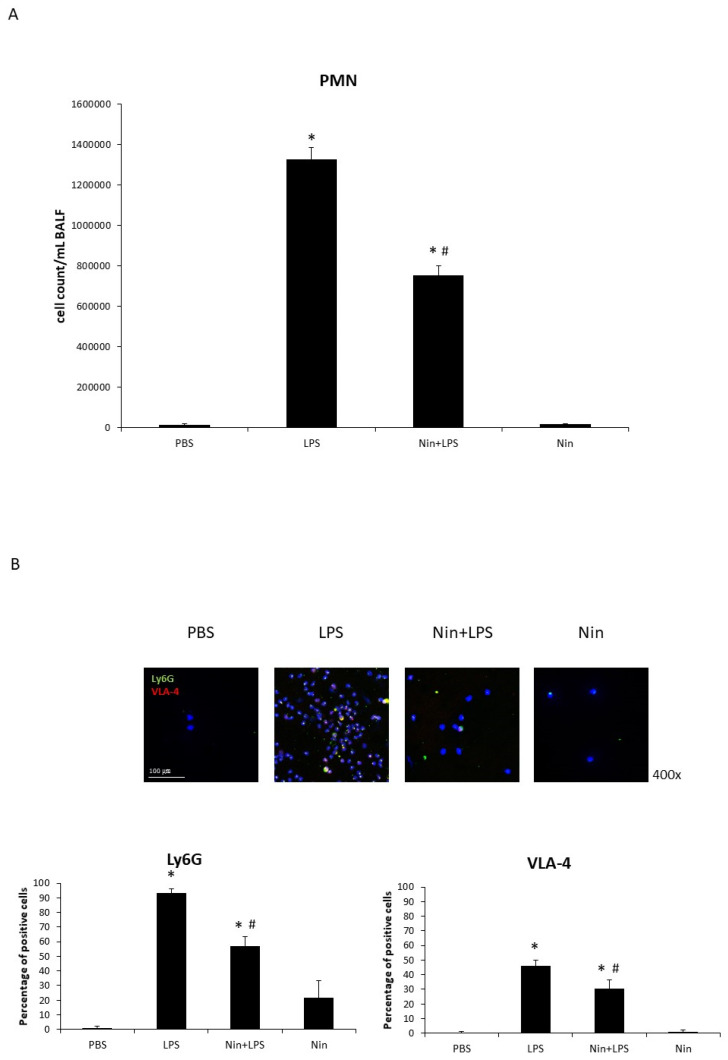
Nintedanib (Nin) administration improved pulmonary polymorphonuclear neutrophil (PMN) recruitment in mice with acute lung injury (ALI). (**A**) The bronchoalveolar lavage fluid (BALF) showed marked accumulation of PMNs in the lungs of mice with LPS-induced ALI compared with control mice. Mice with ALI treated with nintedanib had significantly reduced PMN counts. (**B**) Immunofluorescence (IF) staining showed LPS induced the expression of Ly6G and VLA-4 on neutrophils in the BALF, and nintedanib downregulated this expression. Data are means ± standard deviations. * *p* < 0.05 vs. control, # *p* < 0.05 vs. LPS; *n* = 6 per group. PBS, phosphate-buffered saline.

BALF analysis revealed a marked accumulation of PMNs in the lungs of mice with LPS-induced ALI and significantly reduced PMN numbers in nintedanib-treated mice (PBS, 12,500/mL vs. LPS, 1,325,000/mL vs. Nin + LPS, 752,500/mL; both *p* < 0.05; Figure 4A). Immunofluorescence staining showed increased expression of Ly6G and VLA-4 in BALF neutrophils from mice with LPS-induced ALI relative to the control (93.1% vs. 1.7% and 46.0% vs. 0.8%, respectively; both *p* < 0.05). This expression was significantly downregulated in mice treated with nintedanib (Ly6G, 93.1% vs. 56.8%; VLA-4, 46.0% vs. 30.7%; both *p* < 0.05; Figure 4B).

### 2.4. Effect of Nintedanib on CXCR2 and GRK2 Expression Levels

**Figure 5:**
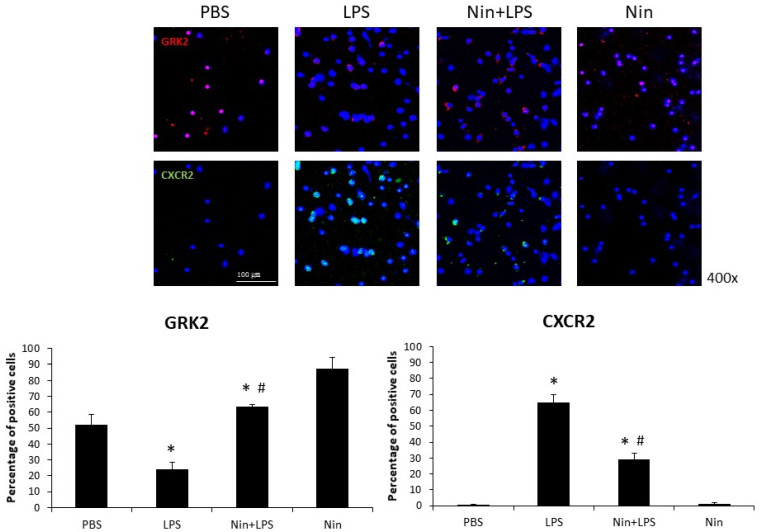
Nintedanib (Nin) administration restored the changes in chemokine (C-X-C motif) receptor 2 (CXCR2) and G protein-coupled receptor kinase 2 (GRK2) expression on circulating neutrophils and prevented pulmonary neutrophil accumulation in mice with LPS-induced ALI. LPS reduced the expression of GRK2 and induced the expression of CXCR2 on circulating neutrophils in mice with LPS-induced ALI, and nintedanib administration restored these changes. Data are means ± standard deviations. * *p* < 0.05 vs. control, # *p* < 0.05 vs. LPS; *n* = 6 per group. PBS, phosphate-buffered saline.

Immunofluorescence staining showed that nintedanib downregulated CXCR2 expression on circulating neutrophils in the peripheral blood of mice with ALI (29% vs. 65%, *p* < 0.05). Confocal microscopy revealed that the expression of GRK2 on circulating neutrophils was significantly upregulated in mice treated with nintedanib compared with that in mice with ALI (63% vs. 24%, *p* < 0.05; Figure 5).

### 2.5. Effects of Nintedanib on Human Neutrophil Migration

**Figure 6:**
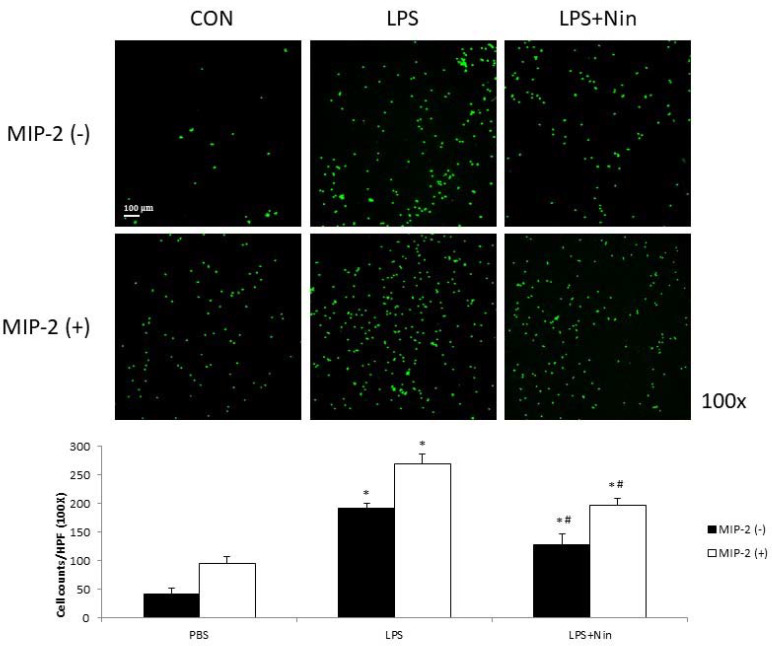
Nintedanib (Nin) reduced human neutrophil migration stimulated by MIP-2 or LPS in a neutrophil migration model. Human neutrophils (2 × 10^5^) isolated from patients with septic shock were placed in upper wells. MIP-2 or LPS enhanced human neutrophil migration. Nintedanib administration reversed these changes. Data are presented as the mean ± standard deviation. * *p* < 0.05 vs. control, # *p* < 0.05 vs. LPS; *n* = 6 per group. PBS, phosphate-buffered saline; HPF, high-power field.

We investigated the effects of nintedanib on human neutrophil migration using the transwell migration model (Figure 6). Human neutrophils isolated from patients with septic shock were placed in upper wells, while chemotactic agents (MIP-2) were placed in lower wells. LPS increased neutrophil migration compared to cells without stimulation (LPS alone, 191 vs. control, 42; *p* < 0.05) and showed an additional effect when combined with MIP-2 (LPS + MIP-2, 270, *p* < 0.05). Migration was reduced for neutrophils treated with LPS + nintedanib (127, *p* < 0.05) compared to neutrophils treated with LPS alone. Reduced neutrophil migration was also observed in neutrophils treated with LPS + MIP-2 + nintedanib (197, *p* < 0.05) compared to neutrophils treated with LPS + MIP-2.

## 3. Discussion

To our knowledge, this study is the first to show that nintedanib regulates neutrophil migration in the setting of ALI. This study offers four major contributions to the literature by showing that: (i) nintedanib administration reduced neutrophil infiltration in the lung, reducing the pathological severity of LPS-induced ALI (Figure 1, Figure 2 and Figure 4); (ii) nintedanib treatment reduced the percentage of fibrotic changes in the lungs of mice with ALI (Figure 1 and Figure 3); (iii) nintedanib downregulated Ly6G and VLA-4 expression levels on neutrophils in BALF (Figure 4); and (iv) nintedanib upregulated GRK2 expression and downregulated CXCR2 expression to reduce neutrophil migration in mice with ALI (Figure 5 and Figure 6).

Studies have investigated the benefits of nintedanib in the treatment of pulmonary fibrosis [9], and our previous work [8] showed that nintedanib reduces the severity of bleomycin-induced pulmonary fibrosis and neutrophil accumulation in the lung. Neutrophil migration into the lung plays a critical role in the acute inflammatory response of ALI [10]. The present report provides the first evidence that nintedanib effectively regulates neutrophil chemotaxis in the setting of ALI. The effects of nintedanib treatment on sepsis-induced ALI were similar to those on pulmonary fibrosis; nintedanib reduced fibrotic changes that occurred in response to LPS stimulation in LPS-induced ALI in vivo. CXCR2 plays a major role in neutrophil migration, and such migration into the lung can be suppressed by the inhibition of CXCR2 in the setting of ALI [11,12]. GRK2 regulates neutrophil migration [5] via the phosphorylation of CXCR2 and desensitization of CXCR2 [13]. In this study, we found that nintedanib restored the elevated white blood cell count, but did not change the total protein concentration, in BALF from mice with LPS-induced ALI. Changes in the expression of adhesion molecules (VLA-4 and VCAM-1) resulted from acute pulmonary neutrophil recruitment in mice with LPS-induced ALI. Nintedanib inhibited neutrophil migration in these mice, as confirmed by the reduced VLA-4 and VCAM-1 activity revealed by IHC staining and Western blot analysis. Furthermore, nintedanib downregulated CXCR2 expression and upregulated GRK2 expression on circulating neutrophils from mice with ALI.

LPS administration was found to induce the up-modulation of CXCR2 [14] and down-modulation of GRK2 [15] on circulating granulocytes, mediated by p38 mitogen-activated protein kinase (MAPK) activation. We previously found that stem cell therapy inhibited neutrophil migration by downregulating p38 activity on circulating neutrophils in mice with LPS-induced ALI [16], and that nintedanib reduced neutrophil chemotaxis to regulate the severity of bleomycin-induced pulmonary fibrosis [8]. Those effects were associated with the enhancement of GRK2 activity and reduction of CXCR2 expression on neutrophils. In this study, we found that nintedanib attenuated neutrophil migration and accumulation in the lung in mice with LPS-induced ALI, in part by enhancing GRK2 activity and reducing CXCR2 expression. These findings reflect the effect of p38 MAPK signaling on sepsis-induced ALI [17]. Many studies have demonstrated the role of p38 MAPK in integrin activation in the setting of acute inflammation [18]. Our findings suggest that nintedanib also has a specific immunomodulatory effect on p38 MAPK activity. CXCR2 activation leads to the activation of the p38 MAPK signaling pathway, and thereby the regulation of cell survival and migration, in inflammatory diseases [19]. Having established that nintedanib downregulates the expression of adhesion molecules by modulating p38 MAPK, we believe that GRK2 and CXCR2 play additional roles in protecting against neutrophil chemotaxis in ALI.

This study has several limitations. The pathophysiology of ALI is complex, involving various types of inflammatory cell and different mechanisms. We have explored the effects of nintedanib on neutrophil chemotaxis; however, these results do not represent all of the effects of nintedanib on inflammation in the setting of LPS-induced ALI. Additional studies focusing on the other immune cells that ameliorate ALI are warranted.

## 4. Materials and Methods

### 4.1. Experimental Animals

Male C57BL/6 mice aged 8–12 weeks were purchased from the National Experimental Animal Center (Taipei, Taiwan) and maintained at the Laboratory Animal Center of Taipei Veterans General Hospital (Taipei, Taiwan). They were kept under a 12 h/12 h light/dark cycle and had access to food and water ad libitum. All experimental procedures followed institutional animal care guidelines and used committee-approved protocols (Taipei Veterans General Hospital IACUC no. 2020-051).

### 4.2. Experimental Design

LPS-induced lung injury in mice is considered to be an experimental model of ALI. A murine model of LPS-induced ALI established in our previous work [16,20,21,22,23,24] was used in this study. Briefly, after anesthesia induction, each mouse received an intratracheal instillation of LPS from *Escherichia coli* (0111:B4; Sigma-Aldrich, St. Louis, MO, USA) at a dose of 5 mg/kg in 50 µL phosphate-buffered saline (PBS). Control mice received intratracheal instillations of 50 µL PBS each. Twenty-four hours and 10 min before LPS instillation, each mouse received nintedanib suspended in 300 µL 0.5% hydroxyethyl cellulose (HEC) at a dose of 50 mg/kg or 300 µL HEC orally in a modification of our previously reported procedure [8]. After 24 h, samples were collected from each mouse for the assessment of ALI via histological, immunohistochemical (IHC), immunofluorescence, and bronchoalveolar lavage fluid (BALF) analyses.

### 4.3. Histological and IHC Analyses

Lung tissue was excised from mice in the nintedanib and control groups 24 h after LPS-induced lung injury. Lung tissues were fixed in 4% paraformaldehyde for 10 min, embedded in paraffin, and cut into 4 µm-thick sections. Staining for lymphocyte antigen 6 complex locus G6D (Ly6G; LS-C36561, 1:100; LifeSpan Biosciences, Seattle, WA, USA), very late antigen 4 (VLA-4; #8440S, 1:1000; Cell Signaling, Danvers, MA, USA), vascular cell adhesion molecule 1 (VCAM-1; #14694, 1:1000; Cell Signaling), and collagen-1 (ab34710, 1:100; Abcam, Cambridge, UK) was performed using Envision^®^ + Dual Link System-HRP (DAB+) kits (K4065; DAKO, Carpinteria, CA, USA). Briefly, the sections were deparaffinized with xylene, dehydrated with ethanol, and then heated in 0.01 M citrate buffer (pH 6.0). Endogenous peroxidase activity was inactivated in 3% H_2_O_2_ for 10 min at room temperature, and the sections were blocked with blocking buffer. Secondary anti-rabbit antibody-coated polymer peroxidase complexes were then applied for 30 min at room temperature, followed by treatment with substrate/chromogen and further incubation for 5–15 s at room temperature. The sections were counterstained with hematoxylin (109249; Merck, Darmstadt, Germany) for 10 s and then washed in running water for 10 min. They were observed and photographed with an Olympus AX80 fluorescence microscope (Olympus America, Melville, NY, USA). The percentage of IHC signal per photographed field (IHC positive area) was determined with image processing software (Image-Pro Plus; Media Cybernetics, Inc., Silver Spring, MD, USA).

### 4.4. Lung Injury Scoring

To histologically quantify the severity of ALI, lung injury scores were calculated. Two investigators independently evaluated each hematoxylin and eosin-stained slide in a blinded manner. To generate the lung injury score, 300 alveoli were counted on each slide at 400× magnification. Within each field, points were assigned according to the criteria used in our previous work [16,20,21,22,23,24]. Scores were calculated using the following formula [25]:

Lung injury score = [(alveolar hemorrhage points/no. of fields) + 2 × (alveolar infiltrate points/no. of fields) + 3 × (fibrin points/no. of fields) + (alveolar septal congestion/no. of fields)]/total number of alveoli counted.

### 4.5. BALF Analysis

The mice were euthanized, and the tracheae were cannulated with a catheter. PBS (0.5 mL) was infused three times into the lungs to collect BALF. PMNs in the BALF were counted using a hemocytometer. The protein contents of the BALF and serum were determined using a bicinchoninic acid protein assay kit (Pierce, Rockford, IL, USA).

### 4.6. Western Blotting

Mouse lung tissues were homogenized in lysis buffer [475 μL RIPA (Thermo, Waltham, MA, USA, M-PER Mammalian Protein Extraction Reagent, REF:78501), 5 μL cocktail (Thermo, Protease Inhibitor Cocktail 100×, 78430), and 20 μL 0.1 M Na_3_VO_4_ (Thermo)], centrifuged at 20,000 rpm and 4 °C for 10 min, and stored at −20 °C until use. Equal amounts of protein homogenate were resolved on 7.5–10% sodium dodecyl sulphate–polyacrylamide electrophoresis gels and transferred to polyvinylidene fluoride membranes. The blots were blocked in Tris-buffered saline with Tween (TBST) containing 5% milk and probed with primary antibodies to VLA-4 (#8440S, 1:1000; Cell Signaling), VCAM-1 (#14694, 1:1000; Cell Signaling), p38 (#9212S, 1:1000; Cell Signaling), phosphorylated (p)-p38 (#9211, 1:1000; Cell Signaling), collagen-1 (ab34710, 1:100; Abcam), and β-actin (20536-1-A, 1:5000; Proteintech, Rosemont, IL, USA). The blots were then washed in TBST, incubated with horseradish peroxidase secondary antibodies [goat anti-rabbit immunoglobulin G (IgG) (H&L), ab6721; Abcam], and detected using an enhanced chemiluminescence substrate (Pierce Biochemicals, Rockford, IL, USA). Each blot was exposed to film, and densitometry of the immunoreactive bands was performed with ImageJ software.

### 4.7. Immunofluorescence (IF) Staining

Cells from BALF and blood were subjected to cytospinning, fixed, permeabilized, and stained with Ly6G (LS-C36561, 1:100; LifeSpan Biosciences), VLA-4 (1:100, ab202969; Abcam), GRK2 (GTX101682, 1:100; GeneTex, Hsinchu City, Taiwan), or Alexa 647-labeled CXCR2 (129101, 1:100; BioLegend, San Diego, CA, USA) antibodies as primary antibodies. On the next day, goat anti-rabbit IgG (H&L) Alexa Fluor^®^ 488 (1:400, ab150077; Abcam), or goat anti-rabbit IgG (H&L) Cy5^®^ (ab6564, 1:400; Abcam) was incubated as a secondary antibody at 37 °C for 2 h. For nuclear staining, the cells were counterstained with DAPI (H-1200; Vector Laboratories, CA, USA). Images of the cells were obtained under a Fluoview confocal microscope (FV10i; Olympus). Five random microscopic fields per well were estimated. IF(+) cells and DAPI(+) cells were counted at 400× magnification. The percentage of positive cells was the ratio of IF(+) cells to DAPI(+) cells.

### 4.8. Isolation of Human Neutrophils

Neutrophils in peripheral blood were obtained from patients with septic shock. All experiments were approved by the institutional review board of Taipei Veterans General Hospital (VGHIRB No. 2019-07-021AC). Consent was obtained from all patients or their surrogates before enrollment.

Peripheral blood was obtained from patients, and neutrophils (purity > 98%) were isolated by plasma-Percoll gradients after dextran sedimentation of erythrocytes [26,27]. Neutrophils were resuspended at a final concentration of 5 × 10^6^ cells/mL in RPMI1640 containing 5% fetal calf serum. Part of neutrophils were cultured with or without 100 ng/mL LPS for 1 h according to the migration assay protocol.

### 4.9. In Vitro Human Neutrophil Migration Assay

Migration assays were conducted in a modified 24-well (3.0-mm) Boyden chamber (BD Biosciences, San Jose, CA, USA). Human neutrophils (2 × 10^5^) were plated in the upper well. Medium containing 1 ng/mL recombinant human MIP-2 (R&D Systems, Minneapolis, MN, USA) was placed in the lower well as a chemotactic stimulus. Nintedanib (400 μmol) was added in the upper well. After 2 h of incubation, the upper surface of the filter was swabbed with cotton to remove nonmigratory cells. Migrated cells were fixed with 10% formalin and stained with DAPI. Five random microscopic fields per well were counted.

### 4.10. Statistical Analysis

To limit variability for each experimental condition, all mice were prepared and studied at the same time. Separate groups of mice were used for lung injury scoring, immunostaining, flow cytometry, and migration assays. The data are presented as means ± standard errors of the mean or means ± standard deviations and were analyzed using Mann–Whitney U tests or Student’s *t* tests when appropriate. *p* values < 0.05 were considered to be significant.

## 5. Conclusions

We have identified the novel role of nintedanib in the attenuation of LPS-induced ALI via the inhibition of neutrophil chemotaxis. This effect was mediated by the upregulation of GRK2 expression and downregulation of CXCR2 expression by nintedanib. These findings provide important evidence that nintedanib has a specific immunomodulatory effect in the setting of ALI and support the future use of this drug for the management of sepsis-induced ALI/ARDS.

## Data Availability

Data available on request from the authors.

## Abbreviations

acute lung injury (ALI), acute respiratory distress syndrome (ARDS), chemokine (C-X-C motif) receptor 2 (CXCR2), polymorphonuclear leukocytes (PMNs), macrophage-inflammatory protein 2 (MIP-2), lipopolysaccharide (LPS), G protein-coupled receptor kinases (GRKs), phosphate-buffered saline (PBS), hydroxyethyl cellulose (HEC), immunohistochemical (IHC), bronchoalveolar lavage fluid (BALF), lymphocyte antigen 6 complex locus G6D (Ly6G), very late antigen 4 (VLA-4), vascular cell adhesion molecule 1 (VCAM-1), Tris-buffered saline with Tween (TBST), nintedanib (Nin), phosphorylated-p38:p38 ratio (P-p38/p38), immunohistochemical stains (IHC), immunofluorescence (IF), high-power field (HPF), mitogen-activated protein kinase (MAPK).
